# *Leishmania* (*Mundinia*) *orientalis* n. sp. (Trypanosomatidae), a parasite from Thailand responsible for localised cutaneous leishmaniasis

**DOI:** 10.1186/s13071-018-2908-3

**Published:** 2018-06-18

**Authors:** Narissara Jariyapan, Teerada Daroontum, Krit Jaiwong, Wetpisit Chanmol, Nuchpicha Intakhan, Sriwatapron Sor-suwan, Padet Siriyasatien, Pradya Somboon, Michelle D. Bates, Paul A. Bates

**Affiliations:** 1 Department of Parasitology, Faculty of Medicine, Chiang Mai University, Chiang Mai, Thailand; 2 Department of Pathology, Faculty of Medicine, Chiang Mai University, Chiang Mai, Thailand; 3 Santisuk Hospital, Santisuk, Nan, Thailand; 4 Department of Parasitology, Faculty of Medicine, Chulalongkorn University, Bangkok, Thailand; 5 Division of Biomedical and Life Sciences, Faculty of Health and Medicine, Lancaster University, Lancaster, UK

**Keywords:** *Leishmania orientalis*, *Mundinia*, Thailand, Cutaneous leishmaniasis

## Abstract

**Background:**

Leishmaniasis is an emerging disease in Thailand with an unknown incidence or prevalence. Although the number of properly characterized and clinically confirmed cases is about 20, it is suspected that this low number masks a potentially high prevalence, with clinical disease typically manifesting itself against an immunocompromised background, but with a substantial number of subclinical or cured cases of infection. To date leishmaniasis in Thailand has been mainly ascribed to two taxa within the recently erected subgenus *Mundinia* Shaw, Camargo & Teixeira, 2016, *Leishmania* (*Mundinia*) *martiniquensis* Desbois, Pratlong & Dedet, 2014 and a species that has not been formally described prior to this study.

**Results:**

A case of simple cutaneous leishmaniasis was diagnosed in a patient from Nan Province, Thailand. Molecular analysis of parasites derived from a biopsy sample revealed this to be a new species of *Leishmania* Ross, 1908, which has been named as *Leishmania* (*Mundinia*) *orientalis* Bates & Jariyapan n. sp. A formal description is provided, and this new taxon supercedes some isolates from the invalid taxon “Leishmania siamensis”. A summary of all known cases of leishmaniasis with a corrected species identification is provided.

**Conclusions:**

Three species of parasites are now known to cause leishmaniasis is Thailand, *L. martiniquensis* and *L. orientalis* n. sp. in the subgenus *Mundinia*, which contains the type-species *Leishmania enriettii* Muniz & Medina, 1948, and a single case of *Leishmania infantum* Nicolle, 1908. This study now enables epidemiological and other investigations into the biology of these unusual parasites to be conducted. It is recommended that the use of the taxonomically invalid name “L. siamensis” should be discontinued.

**Electronic supplementary material:**

The online version of this article (10.1186/s13071-018-2908-3) contains supplementary material, which is available to authorized users.

## Background

Leishmaniasis is an emerging disease in Thailand. The first confirmed autochthonous case was reported in 1999 [1], but since then there have been further cases described that undoubtedly reflect a much higher underlying prevalence of undiagnosed cases and asymptomatic infections, given that no active case detection study has been performed to date and there is little experience in diagnosis of leishmaniasis in Thailand. To date a total of 22 parasitologically confirmed cases have been reported, and of these 19 have been identified using various molecular methods. There has been one case of visceral leishmaniasis due to *Leishmania infantum* Nicolle, 1908 infection [2], but no subsequent reports.

The most frequent parasite found so far is *L. martiniquensis* Desbois, Pratlong & Dedet, 2014, accounting for 15 of the identified cases. This parasite is named after the Caribbean island of Martinique, where it was first isolated [3, 4]. In some of the early Thailand reports this is referred to as “L. siamensis”; however, there are two problems with this usage. The first problem is that “L. siamensis” has never been formally described, so the name is taxonomically invalid (a *nomen nudum*), without any type specimen for reference, and therefore technically should not have been used in any publications. The second problem is subsequent evidence that the group of isolates that have previously been called “L. siamensis” has been found not to be monophyletic, and includes two taxa [5, 6]. One of these includes the aforementioned parasites that appear identical to *L. martiniquensis* and, since this is a valid species, this name takes precedence for these particular isolates. However, in the second taxon there are two isolates of “L. siamensis” that are very similar to each other but different from *L. martiniquensis* based on molecular analysis, the PCM2 Trang strain from a patient in southern Thailand [7], which is phylogenetically distinct [6, 8], and a recent isolate from central Thailand [9]. These two isolates were both from patients that were HIV-infected and presented with disseminated cutaneous leishmaniasis, the former also with visceral disease.

Here we describe a case of simple cutaneous leishmaniasis from northern Thailand that was caused by a parasite apparently very similar to those responsible for these two previous cases, and we formally name this as *Leishmania orientalis* Bates & Jariyapan n. sp., thereby establishing a taxonomically valid name for these parasites, and simultaneously eliminating the need to use the unavailable name “L. siamensis”. We place this new species in the recently erected *Leishmania* subgenus *Mundinia* Shaw, Camargo & Teixeira, 2016 [10] and provide an updated identification of all known previous isolates from Thailand into *L. infantum*, *L. martiniquensis* and *L. orientalis* n. sp.

## Results

### Case report

**Fig. 1:**
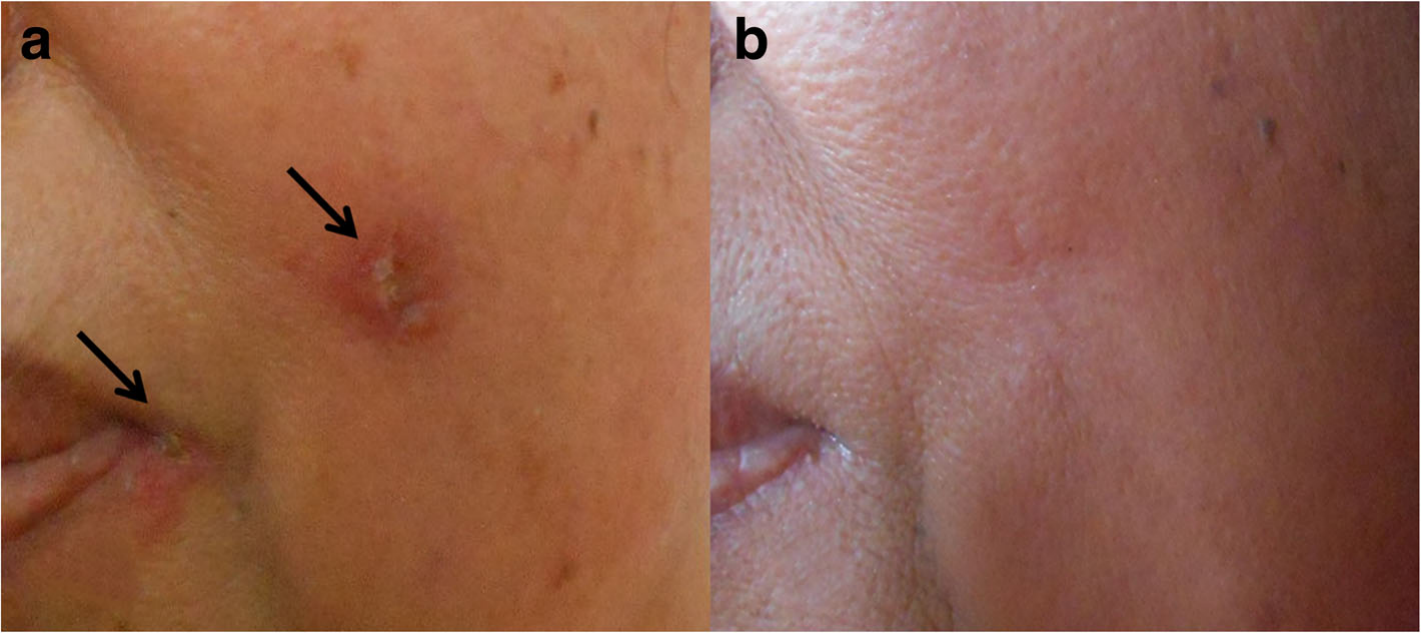
Clinical presentation of cutaneous leishmaniasis. **a** A nodule on the cheek and a crusted sore in the angle of the lips of the patient before treatment, both arrowed. **b** The same view of the patient’s skin after treatment

**Fig. 2:**
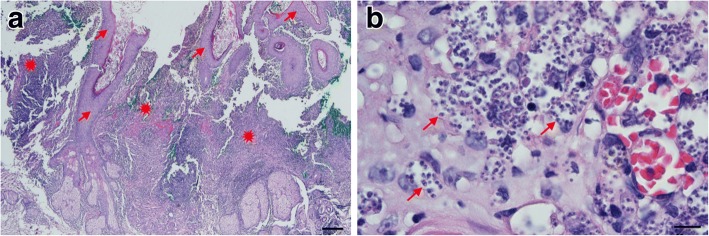
Histopathology of skin biopsy from a nodule on the left cheek (Giemsa stain). **a** Low power magnification photomicrograph showing pseudo-epitheliomatous hyperplasia of the epidermis (arrows) and heavy chronic inflammation of dermis (starbursts). **b** High power magnification photomicrograph showing numerous *Leishmania* amastigotes within the cytoplasm of macrophages and in the extracellular matrix (arrows). *Scale-bars*: **a**, 200 μm; **b**, 10 μm

The patient was a 57-year-old woman who lives in Chiang Klang District, Nan Province, northern Thailand. She is a gardener and has never been abroad, only travelling to Phitsanulok and Phijit, provinces near Nan in Thailand. The patient presented in May 2014 at Chiang Klang Hospital with a single skin nodule on her left cheek (1.0 × 1.5 cm), and also with crusting at the left angle of the mouth (Fig. 1a). No skin nodules in other sites of the body were observed. Two pieces of formalin-fixed skin biopsy from the cheek nodule (0.6 × 0.5 × 0.4 cm^3^ and 0.5 × 0.5 × 0.2 cm^3^) were sent to the Department of Pathology, Faculty of Medicine, Chiang Mai University for histopathological investigation. Histopathological analyses revealed epidermal ulceration with a heavy, chronic inflammation of the dermis (Fig. 2a) and numerous intracellular small round or oval-shaped bodies, with the appearance of amastigotes (1–2 μm in width and 2–4 μm in length) of *Leishmania* spp. (Fig. 2b). A week later, a fresh skin biopsy from the nodule (0.4 × 0.5 × 0.3 cm^3^) was collected and sent to Department of Parasitology, Faculty of Medicine, Chiang Mai University for confirmation of diagnosis by parasite culture and species identification. The skin biopsy sample was cultured in Schneider’s insect medium supplemented with 20% foetal bovine serum (FBS) and 50 International Units penicillin/ml, 50 μg/ml streptomycin at 25°C. Motile promastigotes were first observed on day 3 of the culture. Therefore, the patient was confirmed as diagnosed with cutaneous leishmaniasis. She was treated with oral amphotericin B at 1 mg/kg/day for 1 day and fluconazole at 200 mg/day for 45 days. The skin lesion had disappeared completely by six months after treatment (Fig. 1b). Pre-treatment laboratory investigation showed only mild anaemia with a haemoglobin concentration of 10.9 g/dl, white blood count of 7700 cells/mm^3^, and platelet count of 483,000/mm^3^. There was no hepatosplenomegaly or palpable lymph nodes. Liver function was not investigated, renal function was within normal limits and HIV serology was negative. The patient declined a bone marrow biopsy for evaluation of visceral leishmaniasis. She did not report any other underlying disease, routine drug use, or any other symptoms, and in general was in a good state of health.

### Parasite characterization

**Fig. 3:**
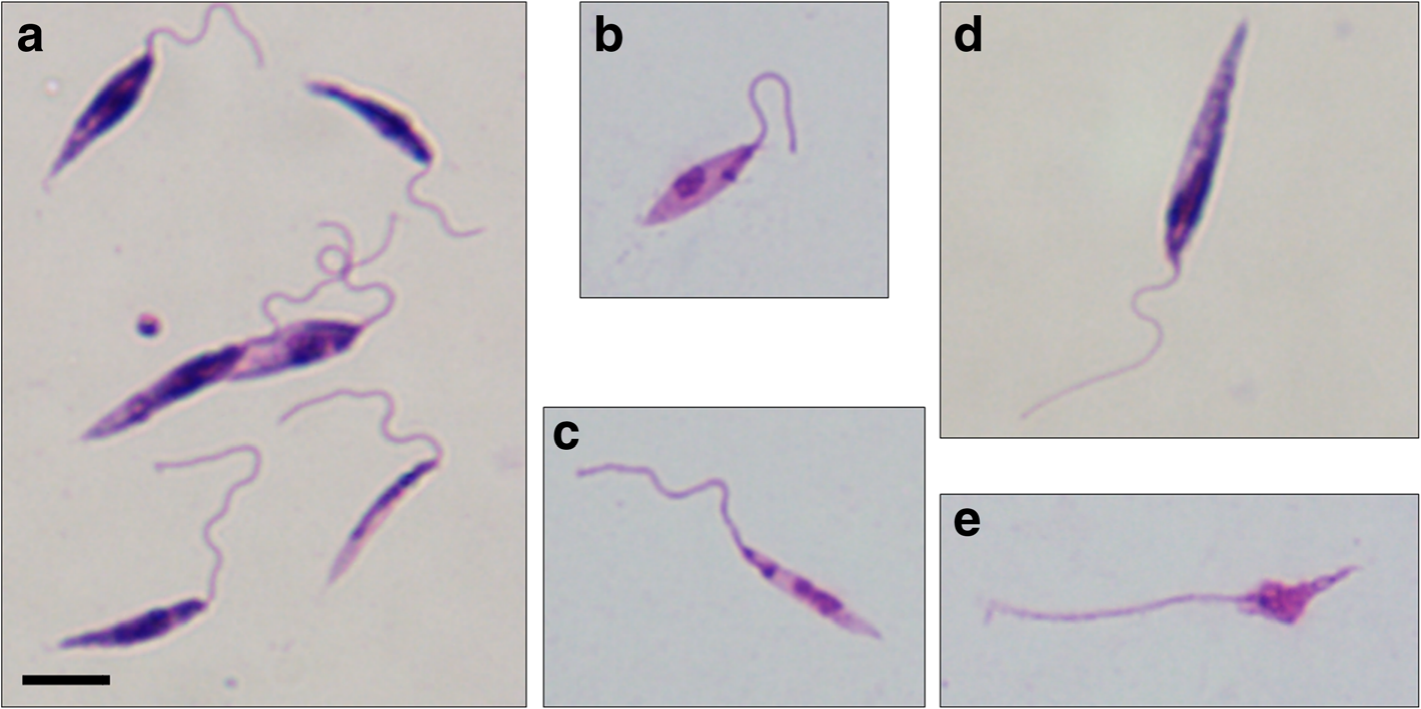
Morphology of Giemsa-stained promastigote forms from culture showing morphological variation of forms observed (**a**); procyclic-like promastigote (**b**); leptomonad-like promastigote (**c**); nectomonad-like promastigote (**d**); and metacyclic-like promastigote (**e**). All images are at the same magnification. *Scale-bar*: 5 μm


The patient isolate has the World Health Organisation strain designation MHOM/TH/2014/LSCM4, hereafter referred to as CM4. Promastigote cultures were established by a serial sub-passage from the initial culture using liquid media, and their morphology examined by light microscopy of Giemsa-stained slides (Fig. 3). The morphological forms observed were generally similar to those described for other *Leishmania* species, demonstrating a range of promastigote types, and including some similar to procyclic promastigotes, leptomonad promastigotes, nectomonad promastigotes and metacyclic promastigotes [11]. Although free-swimming individual promastigotes were readily observed, rosettes and large aggregates of promastigotes were prevalent in culture (Additional file 1: Video S1).

**Additional file 1:** **Video S1.** Live *Leishmania orientalis* promastigotes *in vitro*, illustrating both free-swimming and aggregations of promastigotes. (WMV 28731 kb)


### Molecular analysis

**Fig. 4:**
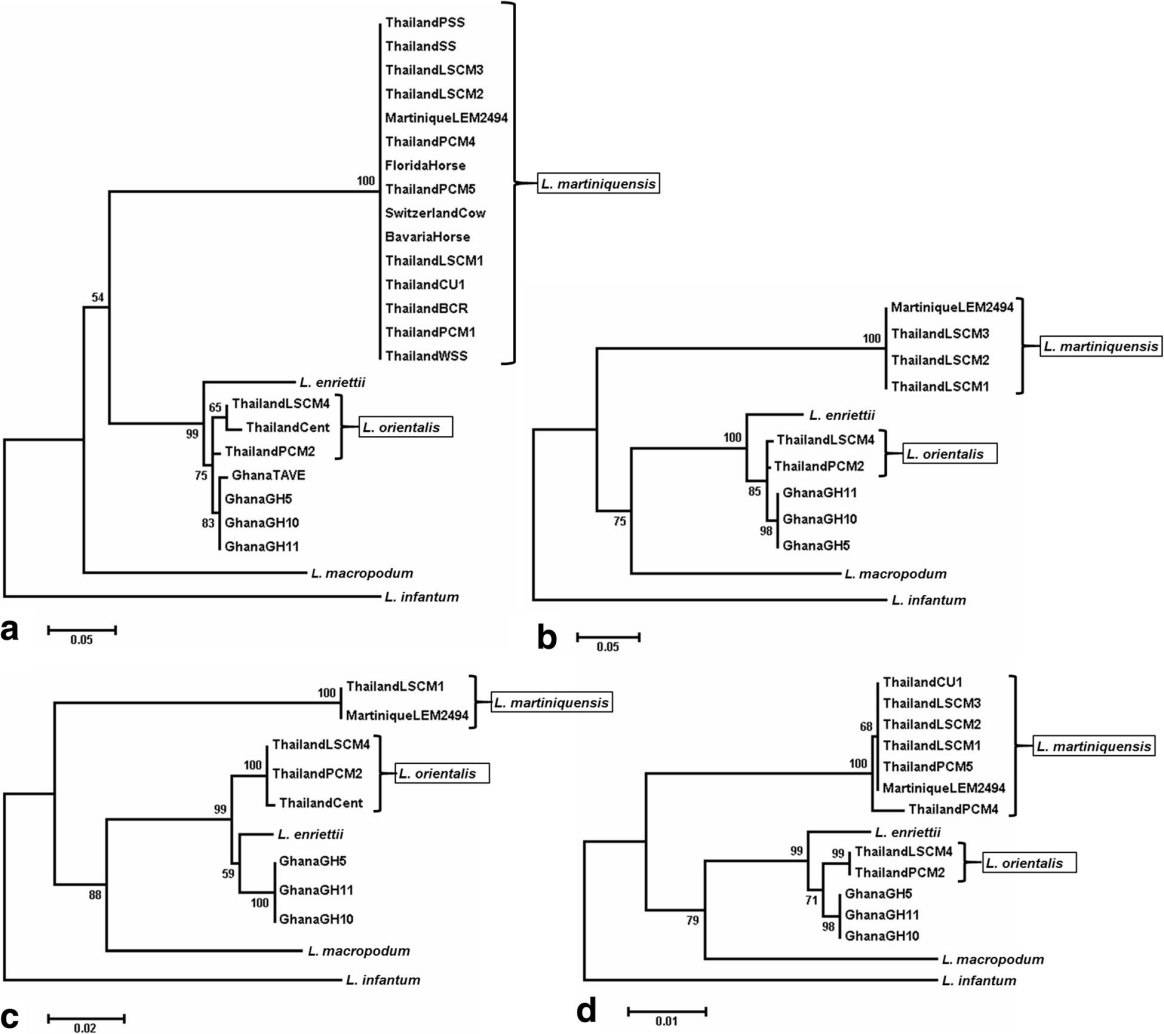
Phylogenetic analysis of DNA sequences from LSCM4 and other members of the subgenus *Leishmania* (*Mundinia*). Each panel shows a maximum likelihood tree with all currently available sequence data from members of the subgenus, using *L. infantum* as an outgroup, and with numbers at nodes derived by bootstrapping at 1000 replicates. **a** ITS1. **b** RPL23a. **c** RNA PolII. **d** HSP70. For accession numbers see Additional file 2: Table S1. The CM4 clade is labelled *L. orientalis*

Four different sequences were analysed by polymerase chain reaction (PCR) and deoxyribonucleic acid (DNA) sequencing: the ribosomal ribonucleic acid (RNA) internal transcribed spacer 1 (ITS1) [12]; the ribosomal protein L23a intergenic sequence (RPL23a) [13]; the large subunit of RNA Polymerase II (RNA PolII) [14]; and heat-shock protein 70 (HSP70) [15]. The accession numbers for the new sequences generated in this study together with others used in the following analyses are given in Additional file 2: Table S1. Initial Basic Local Alignment Search Tool analysis revealed the CM4 sequences to be closest to PCM2 Trang [7] and/or the related isolate from central Thailand [9] for all four sequences examined, and then to other members of the subgenus *Leishmania* (*Mundinia*) (Fig. 4). In three of the analyses the CM4 clade was closest to that containing the recently described (but not yet named) parasites from Ghana [8], and in the RNA PolII tree appeared equidistant between these and *L. enriettii*. In all phylogenetic analyses the CM4 clade (labelled *L. orientalis* in Fig. 4) is clearly distinct from the *L. martiniquensis* clade that also contains several Thai isolates. The sequences of CM4 and PCM2 Trang were very similar to each other or identical in all cases (Table 1), whereas CM4 and a Thai isolate of *L. martiniquensis* called CM1 [6] were different (Table 2).

**Table 1:** Comparisons of LSCM4 DNA sequences with those from PCM2 Trang [7]. The lengths of the sequences given are the number of nucleotides, the analysis excludes PCR primers and comparisons were made using Clustal Omega

Sequence	Length LSCM4	Length PCM2	% identity^a^	No. of identical nt	No. of differences
ITS-1	251	251	96.4	242	9
RPL23a IGS	468	468	99.4	465	3
RNAPolII	1206	1206	100	1206	0
HSP70	1319	1319	100	1319	0

^a^% identity = no. of identical nucleotides/longest length, where sequence length differs

**Table 2:** Comparisons of LSCM4 DNA sequences with those from *L. martiniquensis* LSCM1 [6]. The lengths of the sequences given are the number of nucleotides, the analysis excludes PCR primers and comparisons were made using Clustal Omega

Sequence	Length LSCM4	Length LSCM1	% identity^a^	No. of identical nt	No. of differences
ITS1	251	259	59.1	153	106
RPL23a IGS	468	472	64.8	306	166
RNAPolII	1206	1206	91.5	1104	102
HSP70	1319	1319	95.7	1262	57

^a^% identity = no. of identical nucleotides/longest length, where sequence length differs

### Taxonomic description

**Class Kinetoplastea Honigberg, 1963**

**Order Trypanosomatida Kent, 1880**

**Family Trypanosomatidae Doflein, 1951**

**Genus** ***Leishmania*** **Ross, 1903**

**Subgenus** ***L***. **(*Mundinia*)** **Shaw, Camargo & Teixeira 2016**

***Leishmania*** **(** ***Mundinia*** **)** ***orientalis*** **Bates & Jariyapan n. sp.**

***Type-host*** **:** *Homo sapiens.*

***Type-locality*:** Chiang Klang District (19°17'30"N, 100°51'42"E), Nan Province, Thailand.

***Type-material*:** Hapantotypes, cryopreserved promastigotes stored in liquid nitrogen at the Department of Parasitology, Chiang Mai University, Thailand (accession LSCM4) and the Division of Biomedical and Life Sciences, Lancaster University, UK (accession LV768).

***Strain designation*:** MHOM/TH/2014/LSCM4.

***Homologous strains*:** PCM2 Trang [7] and an unnamed isolate from central Thailand [9].

***Vector*:** Unknown.

***Reservoir(s)*:** Unknown.

***Representative DNA sequences*:** The RNA internal transcribed spacer 1, ribosomal protein L23a intergenic sequence, partial large subunit of RNA Polymerase II and partial heat-shock protein 70 sequences were deposited in the GenBank database under the accession numbers MG731227, MG731231, MG731232 and MG731233, respectively.

***ZooBank registration*:** To comply with the regulations set out in article 8.5 of the amended 2012 version of the *International Code of Zoological Nomenclature* (ICZN) [16], details of the new species have been submitted to ZooBank. The Life Science Identifier (LSID) of the article is urn:lsid:zoobank.org:pub:6C6CBD86-4C7A-437E-B4C7-B6229C9F6C67. The LSID for the new name *Leishmania orientalis* is urn:lsid:zoobank.org:act:C03ADE08-34BD-4252-A534-831D57A3A8D9.

***Etymology*:** Species name refers to eastern origin since this parasite is unlikely to be confined to Thailand only.

### Description (Figs. 2, 3)

***Morphology*.** Amastigotes 1–2 μm in width and 2–4 μm in length. Promastigotes of various sizes with body length ranging between 5–15 μm and motile with a free anterior flagellum of variable length.

***Growth in vitro*.** Isolated in Schneider’s *Drosophila* medium supplemented with 20% (v/v) FBS and maintained in the same medium or Medium 199 supplemented with 10% (v/v) FBS and Basal Medium Eagle (BME) vitamins. Relatively easy to grow compared to other *Leishmania* species by subpassage every 1–2 weeks.

***Pathology*.** Variable; in the type-host presentation was cutaneous leishmaniasis with no known underlying immunodeficiency; in other strains presentation as disseminated cutaneous leishmaniasis, with possible visceral disease, both in the presence of human immunodeficiency virus (HIV) co-infection.

### Remarks

The new species was characterized using molecular techniques, revealing a distinct parasite within the subgenus *Mundinia*. Two isolates, PCM2 Trang [7] and an unnamed isolate from central Thailand [9] are considered as being *L*. (*M*.) *orientalis* due to their high level of similarity with the type-strain LSCM4.

## Discussion

Here we describe a new species of *Leishmania* causing human disease in Thailand, *Leishmania orientalis*. Together with *L. martiniquensis* the new species accounts for almost all of the known cases of leishmaniasis reported in Thailand (Table 3). The only exception is one case of *L. infantum* reported in 2008 [2], but in that case it was suspected the infection may have been acquired elsewhere.

**Table 3:** Reports of leishmaniasis from Thailand and Myanmar ordered by year of isolation. Where done, identification was performed by sequencing one or more DNA targets. Those identified with^a^ were originally or subsequently reported as “*L. siamensis*” but later identified to be *L. martiniquensis* by Pothirat et al. [6]. Those identified with^b^ were originally reported as “*L. siamensis*” but shown in this study to be very similar to *L. orientalis*

Year	Location	Age	Sex	HIV	Primary clinical presentation	Species identification	Reference
1996	Surat Thani, Thailand	3	Female	No	Visceral leishmaniasis	Unknown	Thisyakorn et al. (1999) [1]
2005	Nan, Thailand	40	Male	No	Visceral leishmaniasis	Unknown	Kongkaew et al. (2007) [35]
2006	Phang-Nga, Thailand	55	Male	No	Visceral leishmaniasis	*L. martiniquensis* ^a^	Sukmee et al. (2008) [36]
2007	Bangkok, Thailand	66	Male	No	Visceral leishmaniasis	*L. infantum*	Maharom et al. (2008) [2]
2009	Chantaburi, Thailand	37	Male	Yes	Visceral leishmaniasis	*L. martiniquensis* ^a^	Suankratay et al. (2010) [37]
2010	Trang, Thailand	35	Female	Yes	Disseminated cutaneous and visceral leishmaniasis	*L. orientalis* ^b^	Bualert et al. (2012) [7]
2010	Satun, Thailand	7	Female	No	Visceral leishmaniasis	*L. martiniquensis* ^a^	Osatakul et al. (2014) [38]
2011	Songkhla, Thailand	46	Male	Yes	Visceral and cutaneous leishmaniasis	*L. martiniquensis* ^a^	Chusri et al. (2012) [39]
2011	Trang, Thailand	30	Male	Yes	Disseminated cutaneous leishmaniasis	*L. martiniquensis* ^a^	Chusri et al. (2012) [39]
2011	Lop Buri, Thailand	3	Female	No	Cutaneous leishmaniasis	Unknown	Kattipathanapong et al. (2012) [40]
2012	Yangon, Myanmar	22	Female	No	Asymptomatic	*L. martiniquensis* ^a^	Phumee et al. (2013) [41]
2012	Chiang Rai, Thailand	45	Male	Yes	Disseminated cutaneous leishmaniasis	*L. martiniquensis* ^a^	Phumee et al. (2013) [41]
2012	Yangon, Myanmar	34	Male	Yes	Disseminated cutaneous leishmaniasis	*L. martiniquensis* ^a^	Phumee et al. (2013) [41]
2012	Yangon, Myanmar	60	Male	No	Disseminated cutaneous leishmaniasis	*L. martiniquensis* ^a^	Noppakun et al. (2014) [42]
2012	Ban Thi, Thailand	52	Male	No	Visceral leishmaniasis	*L. martiniquensis*	Pothirat et al. (2014) [6]
2013	Hang Dong, Thailand	48	Male	Yes	Disseminated cutaneous leishmaniasis	*L. martiniquensis*	Chiewchanvit et al. (2015) [33]
2013	Mae Tha, Thailand	38	Male	Yes	Disseminated cutaneous leishmaniasis	*L. martiniquensis*	Chiewchanvit et al. (2015) [33]
2014	Chiang Klang, Thailand	57	Female	No	Cutaneous leishmaniasis	*L. orientalis*	Present study
2017	Kanchanaburi, Thailand	42	Female	Yes	Disseminated cutaneous leishmaniasis	*L. orientalis* ^b^	Supsrisunjai et al. (2017) [9]

Both *L. orientalis* and *L. martiniquensis* are members of the recently described *Leishmania* subgenus *Mundinia*, with the type-species *L. enriettii* [10, 17]. Until recently, this was known as the *L. enriettii* species complex [6, 8, 18–20], but based largely on molecular data these species have emerged as a distinct monophyletic clade. Regarding human pathogenicity, the three other subgenera of *Leishmania* apart from *Mundinia* are relatively uniform, with *L*. (*Leishmania*) (type-species *L. donovani* Laveran & Mesnil, 1903) and *L*. (*Viannia*) (type-species *L. braziliensis* Vianna, 1911) containing mainly human-infective species (with one or two exceptions), whereas the *L*. (*Sauroleishmania*) have never been found in humans - the so-called lizard *Leishmania*. In that respect the known members of *Mundinia*, to which we now add *L. orientalis* n. sp., present a rather eclectic mixture: three species are human pathogens, *L. martiniquensis*, *L. orientalis* n. sp. and *Leishmania* species Ghana; and two species are non-pathogenic to humans, *L. enriettii*, known to infect domestic guinea pigs in Brazil [21, 22], and *L. macropodum* Barratt, Kaufer & Ellis, 2017, known to infect certain species of kangaroo and other macropods in Australia [23, 24]. The two other clades closest to *L. orientalis* n. sp. are *L. enriettii* and *Leishmania* species Ghana. From its known features *L. enriettii* is clearly different from *L. orientalis*, and also, given the differences in clinical presentation [8] and the results of the current and previous molecular analyses [8], *Leishmania* species Ghana also appears to be different from *L. orientalis*. Overall the diversity of hosts and variation in human pathogenicity of the species of *Mundinia* is most likely a reflection of their basal position in *Leishmania* phylogeny [8, 25]. This diversity also supports a supercontinental origin of the subgenus, in which the *Mundinia* appeared before the other subgenera and prior to the breakup of Gondwana. This explains its presence in different continents and mammalian orders.

The vectors of the species of *Leishmania* (*Mundinia*) are not known with certainty, including efforts to incriminate sand flies, the normal vectors of leishmaniasis, although *Lutzomyia monticola* has been suggested as a possible vector for *L. enriettii* [26]. The best evidence is for *L. macropodum*, where unusually the vector appears to be a day-biting midge of the genus *Forcipomyia* [13]. Crucially these midges supported the development of infections beyond the blood meal stage and produced material similar to promastigote secretory gel and metacyclic promastigotes (infective stages). In addition, *L. macropodum* and *L. enriettii* can both develop beyond the blood meal in the experimental midge host *Culicoides sonorensis* Wirth & Jones, 1957, whereas neither can establish in the normally permissive sand fly host *Lutzomyia longipalpis* Lutz & Neiva, 1912 [27]. In contrast, *Leishmania* species in other subgenera show full development in *Lu. longipalpis* [28]. On the other hand, in Thailand there have been reports of *Leishmania* DNA in *Sergentomyia* sand flies [29, 30], suggesting the possible involvement of a new sand fly genus in transmission to humans, which would usually be a species of *Phlebotomus* in Asia. However, dissections were not performed to investigate whether these flies supported infection beyond the blood meal stage, which is required for transmission [31].

The recent emergence of leishmaniasis in Thailand is a worrying development, as there is little experience amongst clinicians and public health professionals in diagnosis, treatment or disease control in the country. This is compounded by the nature of the parasites themselves, which are two novel and therefore poorly understood species, *L. martiniquensis* and *L. orientalis* n. sp. and preliminary evidence suggesting that they may have a different type of vector to that normally associated with transmission. The underlying rate of infection is almost certainly much higher than the number of case reports indicates, and could conceivably be a common but sub-clinical infection in most infected individuals, only manifesting when patients become immunocompromised in some way. In a recent study, the prevalence of *Leishmania* infection in a cohort of HIV patients from Trang Province, Thailand was 25.1% [32]. Whilst these data may not be representative of the whole country, they clearly demonstrate the potential for a high underlying rate of exposure to *Leishmania* spp. in the general population. Clearly more work is required both on the basic biology and vectors of these species, as well as epidemiological investigation of the prevalence and incidence of infection in the human population.

## Methods

### Parasite culture and morphology

Promastigote cultures were obtained by inoculation of patient biopsy material into 5 ml of Schneider’s Drosophila medium (Sigma-Aldrich, St Louis, MO, USA) supplemented with 20% (v/v) FBS (Thermo-Fisher, Waltham, MA, USA) in a 25-cm^2^ tissue culture flask and maintained at 26 °C (Pothirat et al. [6]). Subsequently, promastigotes were passaged in the same medium, and also into Medium 199 (Lonza, Basel, Switzerland) supplemented with 10% (v/v) FBS, BME vitamins (Sigma-Aldrich) and 25 μg/ml gentamicin sulphate (Sigma-Aldrich) for long term maintenance. Promastigotes were cryopreserved in 7.5% (v/v) glycerol, 50% (v/v) fetal bovine serum in culture medium and stored in liquid nitrogen. For morphological characterization, the culture parasites were smeared on microscope slides, air-dried, and fixed with absolute methanol. The samples were stained with Giemsa (1:10 in phosphate buffered distilled water, pH 6.8) for 30 min, washed in running water, and drained dry. All sample slides were observed for *Leishmania* parasites under a light microscope at 1000× magnification using an oil immersion lens.

### Histology

Skin samples were biopsied from a nodule of the left cheek of the patient, subsequently fixed in 10% formaldehyde and then sent to the Department of Pathology, Faculty of Medicine, Chiang Mai University. After processing, paraffin-embedded tissue sections of 5 μm thickness were cut and stained with standard haematoxylin-eosin (H&E). All sample slides were observed for general features and *Leishmania* parasites under a light microscope at 400× (dry lens) and 1000× (oil immersion lens), respectively.

### PCR and DNA sequencing

PCR amplification of the ribosomal spacer ITS1 was performed with LeF/LeR primers[12], the RPL23a gene with BN1/BN2 primers [33], the RNA PolII gene with S1/S2 and S3/S4 primers [6], and the HSP70 gene with HSP70sen/HSP70ant primers [15], as previously described. Control DNA was from *L. infantum* (MCAN/ES/98/LEM-935;JPC;M5), which was used as an outgroup in the phylogenetic analyses. Twelve new sequences were generated as indicated in Additional file 2: Table S1. Amplification was performed with proof-reading DNA polymerase (Qiagen HotStar HiFidelity Polymerase) and products directly sequenced using commercial services. Results were checked for quality using Chromas Lite 2.1.1 (http://technelysium.com.au/).

### DNA sequence analysis

Initial alignments and analyses were performed using Clustal Omega (http://www.ebi.ac.uk/Tools/msa/clustalo/). For phylogenetic analysis, alignment and tree building programmes in Molecular Evolutionary Genetics Analysis (MEGA) version 7 were used [34]. For ITS1 sequences the Kimura 2-parameter model (Invariant) gave the best-fitting model of sequence evolution and was used for tree construction using the maximum likelihood (ML) and neighbour-joining (NJ) methods. For the RPL23a sequences the Kimura 2-parameter model, for the large subunit of RNA polymerase II the Tamura-Nei (Gamma) model, and for the HSP70 the Hasegawa-Kishino-Yano (Gamma) model were used for tree construction using the ML and NJ methods. Bootstrapping was performed on all trees with 1000 replicates.

## Additional files

Additional file 2: **Table S1.** Accession numbers for sequences analysed in this study. New sequences are indicated by *. (DOCX 15 kb)

## Abbreviations

BME: Basal Medium Eagle
DNA: Deoxyribonucleic acid
FBS: Foetal bovine serum
H&E: Haematoxylin-eosin
HIV: Human immunodeficiency virus
HSP70: Heat-shock protein 70
ITS1: Internal transcribed spacer 1
MEGA: Molecular Evolutionary Genetics Analysis
ML: Maximum likelihood
NJ: Neighbour-joining
PCR: Polymerase chain reaction
RNA PolII: Large subunit of RNA Polymerase II
RNA: Ribonucleic acid
RPL23a: Ribosomal protein L23a intergenic sequence
