# Supplementary material for: Leishmania (Mundinia) orientalis n. sp. (Trypanosomatidae), a parasite from Thailand responsible for localised cutaneous leishmaniasis
Source: Parasit Vectors. 2018 Jun 18;11:351. doi: 10.1186/s13071-018-2908-3 (PMC6006788; doi:10.1186/s13071-018-2908-3)
Supplement: Supplementary file 2 — Table S1. Accession numbers for sequences analysed in this study. New sequences are indicated by *. (DOCX 15 kb) [file 13071_2018_2908_MOESM2_ESM.docx]

**Additional file 2: Table S1** Accession numbers for sequences analysed in this study. New sequences are indicated by *.

| Species | Isolate | DNA Sequence | | | |
| --- | --- | --- | --- | --- | --- |
|  |  | ITS-1 | RPL23a | RNAPolII | HSP-70 |
| *L. orientalis* | Thailand LSCM4 | MG731227* | MG731231* | MG731232* | MG731233* |
|  | Thailand PCM2 | JX195640 | KP025944 | KM820664 | KC202880 |
|  | Thailand Cent | KX347438 | n.a. | KX347439 | n.a |
|  |  |  |  |  |  |
| *Leishmania* from Ghana | Ghana GH5 | KP006688 | KP006691 | KP054394 | MG731234* |
|  | Ghana GH10 | KP006689 | KP006692 | KP054395 | MG731235* |
|  | Ghana GH11 | KP006690 | KP006693 | KP054396 | MG731236* |
|  | Ghana TAVE | EF524071 | n.a | n.a. | n.a. |
|  |  |  |  |  |  |
| *L. enriettii* | LV90 | KM677932 | FR693773 | AF151727 | MG731237* |
|  |  |  |  |  |  |
| *L. macropodum* | AM-2004 | AY495830 | FR693774 | HM775497 | MG731238* |
|  |  |  |  |  |  |
| *L. martiniquensis* | Martinique LEM2494 | KM677931 | KP025945 | KM820663 | KP244365 |
|  | Thailand LSCM1 | MG731228* | KP244362 | KM677933 | KP244366 |
|  | Thailand LSCM2 | MG731229* | KP244363 | n.a. | KP244367 |
|  | Thailand LSCM3 | MG731230* | KP244364 | n.a. | KP244368 |
|  | Thailand CU1 | JQ001751 | n.a. | n.a. | JX852709 |
|  | Thailand PCM4 | JX195637 | n.a. | n.a. | KC202882 |
|  | Thailand PCM5 | JQ001752 | n.a. | n.a. | KC202881 |
|  | Thailand PCM1 | EF200012 | n.a. | n.a. | n.a. |
|  | Thailand PSS | KU050856 | n.a. | n.a. | n.a. |
|  | Thailand SS | KU050860 | n.a. | n.a. | n.a. |
|  | Thailand WSS | KU050863 | n.a. | n.a. | n.a. |
|  | Thailand BCR | GQ226034 | n.a. | n.a. | n.a. |
|  | Florida Horse | JQ617283 | n.a. | n.a. | n.a. |
|  | Switzerland Cow | GQ281282 | n.a. | n.a. | n.a. |
|  | Bavaria Horse | GQ281278 | n.a. | n.a. | n.a. |
|  |  |  |  |  |  |
| *L. infantum* | JPC M5 | GQ332359 | FR796438 | XM 001467548 | XM 003392632 |
